# Systemic inflammatory response index as a predictor of stroke-associated pneumonia in patients with acute ischemic stroke treated by thrombectomy: a retrospective study

**DOI:** 10.1186/s12883-024-03783-0

**Published:** 2024-08-15

**Authors:** Feng Zheng, Wen Gao, Yinfeng Xiao, Xiumei Guo, Yu Xiong, Chunhui Chen, Hanlin Zheng, Zhigang Pan, Lingxing Wang, Shuni Zheng, Chuhan Ke, Qiaoling Liu, Aihua Liu, Xinyue Huang, Weipeng Hu

**Affiliations:** 1 Department of Neurosurgery, the Second Affiliated Hospital, Fujian Medical University, No.34 North Zhongshan Road, Quanzhou, 362000 Fujian Province China; 2 Neuromedicine Center, the Second Affiliated Hospital, Fujian Medical University, No.34 North Zhongshan Road, Quanzhou, 362000 Fujian Province China; 3 Department of Neurology, the Second Affiliated Hospital, Fujian Medical University, No.34 North Zhongshan Road, Quanzhou, 362000 Fujian Province China; 4 Department of Clinical Laboratory, the Second Affiliated Hospital, Fujian Medical University, No.34 North Zhongshan Road, Quanzhou, 362000 Fujian Province China; 5 Division of Public Management, the Second Affiliated Hospital, Fujian Medical University, No.34 North Zhongshan Road, Quanzhou, 362000 Fujian Province China; 6 Beijing Neurosurgical Institute, Beijing Tiantan Hospital, Capital Medical University, No. 119, South Fourth Ring West Road, Fengtai District, Beijing, 100070 China; 7 Department of Neurosurgery, Ningxia Hui Autonomous Region People’s Hospital, Yinchuan, 750000 China

**Keywords:** Systemic inflammatory response index, Stroke-associated pneumonia, Predictor, Epidemiology

## Abstract

**Background:**

The predictive value of systemic inflammatory response index (SIRI) for stroke-associated pneumonia (SAP) risk in patients with acute ischemic stroke (AIS) treated by thrombectomy remains unclear. This study aimed to investigate the predictive value of SIRI for SAP in patients with AIS treated by thrombectomy.

**Methods:**

We included AIS patients treated by thrombectomy between August 2018 and August 2022 at our institute. We used multivariate logistic regression to construct the prediction model and performed a receiver operating characteristic curve analysis to evaluate the ability of SIRI to predict SAP and constructed a calibration curve to evaluate the prediction accuracy of the model. We evaluated the clinical application value of the nomogram using decision curve analysis.

**Results:**

We included 84 eligible patients with AIS in the analysis, among which 56 (66.7%) had SAP. In the univariate analysis, there were significant differences in sex (*p* = 0.035), National Institute of Health Stroke Scale score at admission ≥ 20 (*p* = 0.019) and SIRI (*p* < 0.001). The results of multivariable logistic analysis showed that the risk of SAP increased with the SIRI value (OR = 1.169, 95% CI = 1.049–1.344, *p* = 0.014). Age ≥ 60 (OR = 4.076, 95% CI = 1.251–14.841, *p* = 0.024) was also statistically significant. A nomogram with SIRI showed good prediction accuracy for SAP in AIS patients treated by thrombectomy (C-index value = 0.774).

**Conclusions:**

SIRI is an independent predictor for SAP in patients with AIS treated by thrombectomy. A high SIRI value may allow for the early identification of patients with AIS treated by thrombectomy at high risk for SAP.

## Background

Acute ischemic stroke (AIS) is a common cause of disability and death worldwide [1, 2] and its two therapeutic options focus on reperfusion with intravenous thrombolysis and endovascular thrombectomy ( EVT ) [3, 4]. Stroke-associated pneumonia (SAP) is a common medical complication of stroke, with an incidence of 7–38% in AIS patients occurring most frequently in the first seven days after a stroke, which may exacerbate the disease, prolong hospital stays, and increase social and economic burdens [5–7]. EVT is a common and effective method for the treatment of AIS. SAP remains a threat to AIS patients, even if they use EVT. Therefore, early identification of SAP is essential for the timely treatment of AIS after EVT [8, 9].

Recently, increasing evidence has shown that inflammatory biomarkers, such as neutrophil-to-lymphocyte ratio, systemic immune-inflammation index and platelet-to-lymphocyte ratio, are associated with SAP [10–13]. While these biomarkers have shown promise, the systemic inflammatory response index (SIRI) offers a unique advantage. The systemic inflammatory response index (SIRI), calculated based on the number of inflammatory cells in the peripheral blood, can comprehensively reflect the balance between the inflammatory response and immune status [14, 15]. SIRI has a good predictive value for the prognosis of some brain tumors [16] and aneurysmal subarachnoid hemorrhage [17, 18]. In addition, SIRI can also predict SAP in AIS patients treated conservatively [15]. However, the predictive value of SIRI for SAP risk in patients with AIS treated by thrombectomy remains unclear. Therefore, this study aimed to investigate the relationship between SIRI and SAP in AIS patients treated by thrombectomy. This would help in the early identification of high-risk patients and ensure that early intervention is implemented to improve prognosis.

## Methods

### Patient selection

We included consecutive AIS patients who underwent thrombectomy at the Second Affiliated Hospital of Fujian Medical University within 6 h of symptom onset between August 2018 and August 2022. The inclusion criteria were: (1) patients diagnosed with ischemic stroke by digital subtraction angiography or cerebral computed tomographic angiography and who underwent thrombectomy within 6 h of symptom onset; (2) patients aged ≥ 18 years; and (3) patients with blood parameters measured within one day after admission, including count of lymphocyte, neutrophil and monocyte. The exclusion criteria were: (1) presence of other diseases, including hematologic disorders, malignant tumors, use of immunosuppressive drugs, active infections within two weeks before admission, and those diagnosed with pulmonary infections at the time of admission, and (2) missing blood parameter data.

This study is based on the current version of the Helsinki Declaration and TRIPODreporting guidelines [19], and all procedures were carried out in accordance with relevant guidelines and regulations. This study was approved the medical ethics committee of the Second Affiliated Hospital of Fujian Medical University, China (Ethical approval no. 533/2022). All procedures conducted in studies involving human participants complied with the ethical standards of the Institutional Research Committee. Since data were evaluated retrospectively, pseudonymously, and was solely obtained for treatment purposes, a requirement of informed consent was waived by the Ethics Committee of the Second Affiliated Hospital of Fujian Medical University, China (Ethical approval no. 533/2022).

### Parameter acquisition

We collected demographic and clinical characteristics from the medical record database of our institute. The SIRI was calculated as follows: SIRI = (neutrophil count × monocyte count) / lymphocyte count [15]. According to previous research [20, 21], patients’ fasting blood was collected within the first day of admission, and their blood parameters were measured, including white blood cell, platelet, neutrophil, hemoglobin, lymphocyte, red blood cell, and monocyte counts. The National Institute of Health Stroke Scale (NIHSS) and Glasgow Coma Scale (GCS) scores were evaluated by trained neurologists upon admission.

### Outcome measure

The outcome of this study was SAP, which occurred most frequently in the first seven days after a stroke. According to the modified Centers for Disease Control and Prevention criteria, the medical diagnosis of SAP is based on radiographic images, clinical signs, and laboratory parameters of pulmonary infection [22].

### Statistical analyses

We used the statistical package for the social sciences software (26th edition, IBMRSPSS R, Chicago, Illinois) for analyses. Continuous variables with normal distribution were expressed as mean ± standard deviation, while other variables were expressed as the median and interquartile range (IQR). We compared nominal variables using a Pearson’s chi-square test or Fisher’s exact test, and continuous variables based on data distribution using the Mann-Whitney U test. Logistic regression analysis was used to determine predictors of postoperative pneumonia after undergoing thrombectomy. We performed univariate logistic regression for each variable. Variables with *p* < 0.2 in univariate analyses were used as input data for the multivariate logistic regression model. We used receiver operating characteristic (ROC) curve analyses to determine the area under the curve (AUC) values. Sensitivity, specificity, and the optimal test cut-off points were determined by calculating the Youden index (sensitivity + [1-specificity]). In addition, the nomogram was formulated based on these predictors in the multivariate analysis using the package “rms” in R (version 3.5.2). The consistency index (equivalent to the AUC value, expressed as the c index) reflects the ability of the SIRI multivariate model to distinguish patients with or without pneumonia. A calibration curve was constructed to evaluate the model’s prediction accuracy by comparing the prediction probability with the observation probability. The calibration curve was considered appropriate if the point on the calibration plot was close to a 45 ° diagonal. We used decision curve analysis to quantify the net benefits under different thresholds to evaluate the clinical effectiveness of the SAP nomogram in a cohort of patients with AIS treated by thrombectomy. Unless otherwise stated, *p* < 0.05 was considered statistically significant.

**Table 1:** Comparison of baseline characteristics of AIS patients with and without SAP

Variables	Non-SAP(*n* = 28)	SAP(*n* = 56)	*p*-value
Demographic characteristic			
Age, years, mean ± SD	60.82 ± 13.54	66.13 ± 12.65	0.081
Age ≥ 60	14(50%)	39(69.6%)	0.079
Sex, female, n(%)	14(50%)	15(26.8%)	0.035
Hypertension, n(%)	16(57.1%)	40(71.4%)	0.19
Diabetes, n(%)	7(25%)	18(32.1%)	0.5
Hyperlipidemia, n(%)	7 (25%)	10(17.9%)	0.442
Coronary heart disease, n(%)	5(17.9%)	8(14.3%)	0.67
Atrial fibrillation, n(%)	9(32.1%)	22(39.3%)	0.522
Smoking history, n(%)	4(14.3)	5(8.9%)	0.708
Previous stroke Type			
Ischemic stroke, n(%)	5(17.9%)	4(7.1%)	0.262
Hemorrhagic stroke, n(%)	0(0%)	2(3.6%)	0.55
Clinical characteristics			
Hospitalization duration, days median[IQR]	10.74[8.34–17.68]	13.91[6.43–20.49]	0.489
coma at admission, n(%)	4(14.3%)	16(28.6%)	0.239
NIHSS at admission, median[IQR]	13.5[12–19.75]	19.5[14–24.75]	0.021
NIHSS at admission ≥ 20	7(25%)	29(51.8%)	0.019
GCS at admission			0.076
3–8	10(35.7%)	32(57.1%)	
9–12	12(42.9%)	20(35.7%)	
13–15	6(21.4%)	4(7.1%)	
Mechanical ventilation, n(%)	2(7.1%)	12(21.4%)	0.178
Gnesthesia methods			0.072
local anesthesia	28(100.0%)	18(89.3%)	
general anesthesia	0 (0.0%)	10(10.7%)	
Oclusion site			0.091
anterior circulation	25(89.3%)	41(73.2%)	
posterior circulation	3 (10.7%)	15(26.8%)	
Laboratory parameters			
Leukocyte, 10^9^/L, median[IQR]	9.50[7.24–14.83]	12.06[10–16.53]	0.031
Neutrophils, 10^9^/L, median[IQR]	7.21[4.69–12.72]	10.47[8.09–14.85]	0.013
Monocytes, 10^9^/L, median[IQR]	0.46[0.29–0.78]	0.65[0.45–0.91]	0.026
Lymphocytes, 10^9^/L, median[IQR]	1.21[0.83–1.69]	1.02[0.63–1.32]	0.07
Platelet, 10^9^/L, median[IQR]	209[176.5–244.5]	190[169.25–245.25]	0.375
Erythrocyte, 10^12^/L, median[IQR]	4.06[3.81–4.6]	4.19[3.78–4.58]	0.761
Hemoglobin, g/dL, median[IQR]	116[112.25–140]	125[111.25–136.25]	0.608
SIRI, 10^9^/L, median[IQR]	2.54[1.84–6.94]	7.79[4.42–14.88]	< 0.001

AIS: acute ischemic stroke; GCS: Glasgow Coma Scale; NIHSS: National Institute of Health Stroke Scale; SAP: stroke-associated pneumonia; SIRI: systemic inflammatory response index

## Results

Of all the patients with AIS screened at our institute between August 2018 and August 2022, 112 were admitted within 6 h of symptom onset and treated by thrombectomy. Patients with missing blood cell count data (*n* = 1), malignant tumors (*n* = 2), and active infection within two weeks of admission (*n* = 25) were excluded. Finally, 84 qualified patients were included in the study (mean age: 64.36 years; female sex: 29 (34.5%)). Fifty-six (66.7%) of the patients with AIS also had SAP. The baseline characteristics of the patients with and without SAP are shown in Table 1. Patients with SAP were older, their NIHSS score at admission was higher, their GCS score at admission was lower, more were male, more had hypertension and more required mechanical ventilation. The incidence of NIHSS ≥ 20 and GCS score (3–8) at admission were higher in patients with SAP than those in patients without SAP. Leukocyte, neutrophil, and monocyte counts were higher, and lymphocyte counts were lower in patients with SAP than in those without SAP. The median SIRI value was 7.79 [4.42–14.88] in patients with SAP, which was significantly higher than that in patients without SAP at 2.54 [1.84–6.94] (*p* < 0.05) (Table 1).

We used multivariate logistic regression analyses to determine the factors suitable for predicting SAP. After further adjusting for confounding factors, the results of multivariate analysis showed that the risk of SAP increased with the SIRI value (OR = 1.169, 95% CI = 1.049–1.344, *p* = 0.014). In addition, significant differences were observed in age (OR = 4.076, 95% CI = 1.251–14.841, *p* = 0.024) (Table 2).

**Table 2:** Multivariable logistic regression of the independent predicting factors of SAP in AIS patients

Variables	Univariate analysis		Multivariate analysis		
	*p*-value		*p*-value	OR	95% CI
Age ≥ 60	0.082		0.024*	4.076	1.251–14.841
Sex, female	0.038		0.061	3.259	0.982–12.003
Hypertension	0.193		0.839	1.130	0.339–3.641
NIHSS at admission ≥ 20	0.022		0.767	1.294	0.294–5.269
GCS at admission			0.474		
3–8	Reference				
9–12	0.205		0.324	0.519	0.137–1.912
13–15	0.034		0.275	0.366	0.055–2.157
Mechanical ventilation	0.797		0.779	0.782	0.122–6.558
Occlussion site	0.102		0.751	1.303	0.266–7.664
SIRI	0.004		0.014*	1.169	1.049–1.344

AIS: acute ischemic stroke; CI: confidence interval; GCS: Glasgow Coma Scale; NA: not applicable; NIHSS: National Institute of Health Stroke Scale; OR: odds ratio; SAP: stroke-associated pneumonia; SIRI: systemic inflammatory response index. Variables with *p* < 0.20 in univariate analysis were included in multivariable logistic regression models for adjustment; *p < 0.05. Multivariable logistic regression was adjusted for age ≥ 60 years, sex, hypertension, mechanical ventilation, admission NIHSS score ≥ 20, GCS score at admission, occlussion site and SIRI

**Fig. 1:**
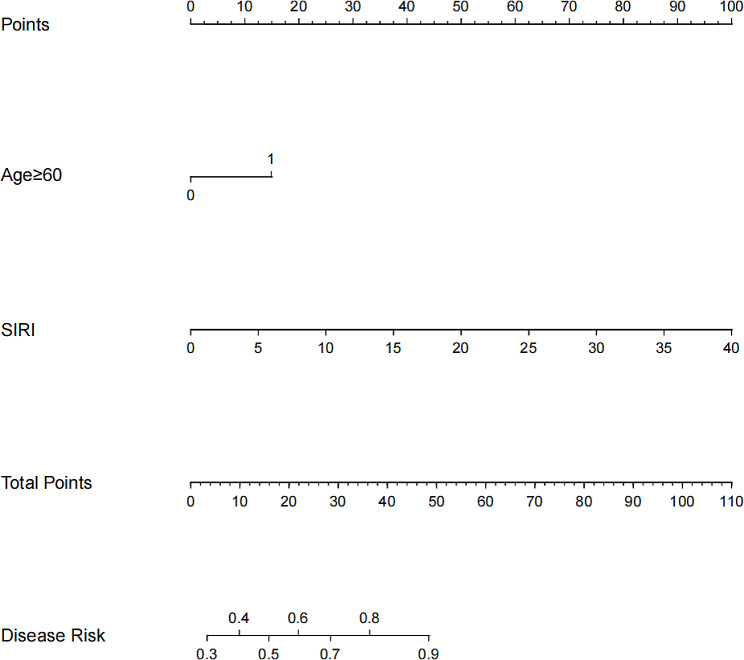
Nomogram for predicting SAP in patients with AIS treated by thrombectomy. The risk nomogram is based on sex, age, and SIRI values. The total score of each variable corresponds to a probability of pneumonia. AIS: acute ischemic stroke; SAP: stroke-associated pneumonia; SIRI: systemic inflammatory response index

**Fig. 2:**
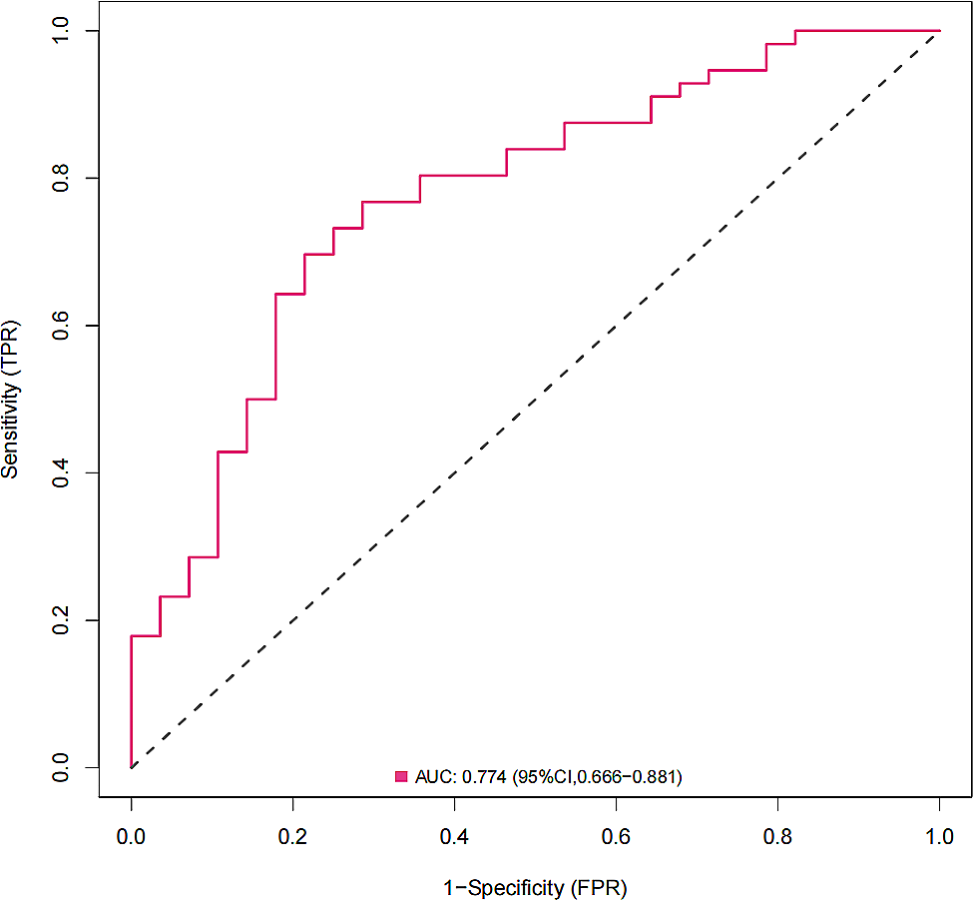
ROC curve of the multivariate SIRI regression model, representing prediction accuracy. ROC: receiver operating characteristic; SIRI: systemic inflammatory response index

**Fig. 3:**
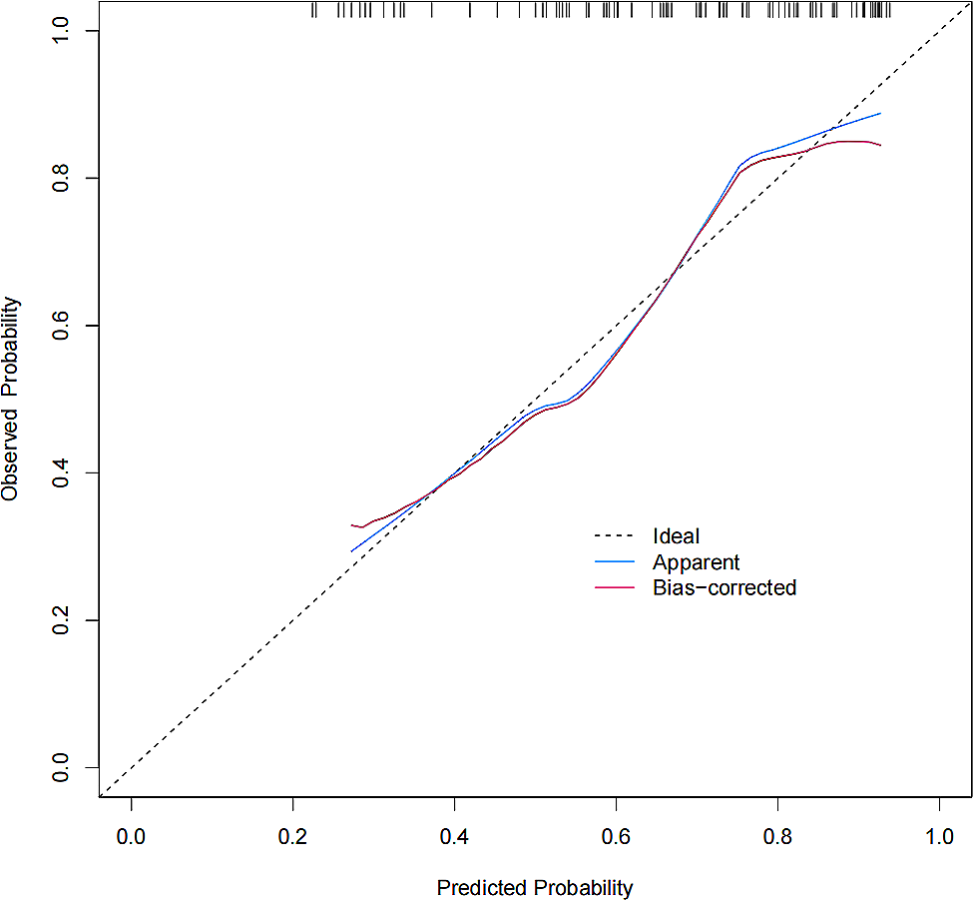
Calibration curve of nomogram predicting SAP in patients with AIS treated by thrombectomy. Note: The prediction of SAP is presented on the X-axis and the actual SAP is presented on the Y-axis. The thick dotted line symbolizes an excellent prediction with an ideal model. The solid line epitomizes the performance of our prediction mode, and the thin dotted line typifies the performance of our optimized model. When the thin dotted line is closer to the thick dotted line, the model is more accurate. When the c index is closer to 1, the accuracy of the nomogram for the risk of SAP in hospitalized patients is higher. AIS: acute ischemic stroke; SAP: stroke-associated pneumonia

**Fig. 4:**
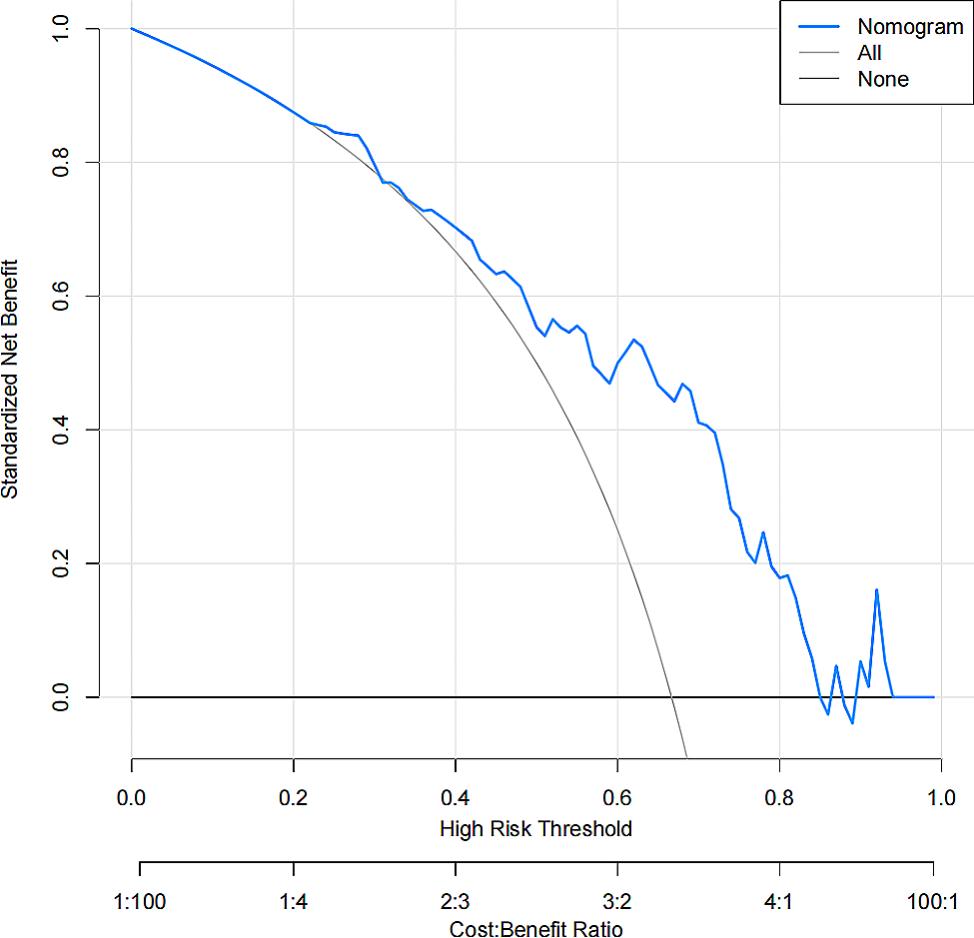
Decision curve analysis of a nomogram for assessing the risk of SAP in patients with AIS treated by thrombectomy. The Y-axis represents the net benefit. The grey line indicates all patients with pneumonia. The black line indicates no pneumonia. The red solid line indicates the risk of pneumonia in the prediction model. AIS: acute ischemic stroke; SAP: stroke-associated pneumonia

Based on two identified variables (age ≥ 60 and SIRI value), we used multivariate logistic regression to construct a predictive model which was shown as a nomogram (Fig. 1). We subsequently analyzed the ROC curve to determine the ability of SIRI values to predict SAP in patients with AIS treated by thrombectomy (Fig. 2). Through the ROC curve of the multivariate model, SIRI could predict SAP in patients with AIS treated by thrombectomy (AUC = 0.774, 95% CI = 0.666–0.881). The optimal critical value of SIRI was 3.617; that is, patients with a score > 3.617 were more likely to develop pneumonia (Youden index = 0.464, sensitivity = 82.1%, specificity = 64.3%). The calibration curve was used to assess the risk of SAP in patients with AIS treated by thrombectomy, with good consistency throughout the cohort, and the data points were close to the 45° diagonal (Fig. 3). The clinical application value of the nomogram was evaluated using the decision curve analysis. According to the decision curve (Fig. 4), within the threshold interval of 0–1, SAP prediction using this model is more profitable.

## Discussion

Acute ischemic stroke (AIS) is a common cause of disability and death worldwide, especially in cases of large vascular occlusions requiring thrombectomy [1, 2] SAP worsens stroke outcomes, lengthens hospitalization, and increases the occurrence of severe disabilities and mortality [5–7]. Therefore, early identification of effective predictors of SAP is critical for timely treatment. To the best of our knowledge, this is the first study to determine the prognostic role of SIRI in the occurrence and progression of SAP in patients with AIS treated by thrombectomy.

In this retrospective single-center study, we analyzed the data of 84 patients with AIS treated by thrombectomy, of which SAP occurred in 66.7%. We found that higher SIRI, the male sex, and age > 60 years were risk factors for SAP [23, 24]. In previous studies, a history of smoking, stroke severity, level of consciousness, high blood pressure, diabetes mellitus, and atrial fibrillation were deemed to be potential predictors of SAP, but similar results were not detected in the present study. This may be due to the relatively small sample size of our study. In addition, in previous reports, age was independent predictors of SAP in patients with AIS, with higher rates of SAP occurring and those aged > 60 years. This is consistent with our findings. Furthermore, in our study, SIRI levels were significantly higher in SAP patients with AIS treated by thrombectomy than in non-SAP patients with AIS treated by thrombectomy (OR = 1.171, 95% CI = 1.034–1.325, *p* = 0.013), indicating that SIRI might be an independent predictor of SAP in patients with AIS treated by thrombectomy. In addition, the optimal critical value of SIRI was 3.617, and prevention and treatment are recommended for patients with SIRI > 3.617.

Nomograms are widely used to predict the possibility of clinical events by integrating various variables. In the present analysis, we constructed a reliable and convenient nomogram to predict SAP in patients with AIS treated by thrombectomy. By integrating demographic characteristics, clinical symptoms, and serum biological indicators, our study developed a nomogram using SIRI. This tool assigns points on a scale to each predictor value and the total points indicate a predicted risk of SAP in patients with AIS who underwent thrombectomy. Our findings suggest that the nomogram has high predictive accuracy and can assist clinicians in making timely decisions for the management of these patients.

The close relationship between SIRI and SAP may be due to stroke-induced immunosuppression. A prolonged excessive inflammatory response depletes the immune system, ultimately suppressing systemic immunity to protect the brain [25]. However, this makes the body more vulnerable to pathogens, leading to stroke-induced immunosuppression syndrome (SIDS) and infections [25, 26]. SIDS is associated with the activation of the sympathetic nervous system (SNS) [27], parasympathetic nervous system (PNS) [28], and hypothalamic-pituitary-adrenal (HPA) axis [29]. Stroke initially overstimulates the SNS, releasing catecholamines (epinephrine, norepinephrine, and dopamine) into the bloodstream [30]. Persistent high catecholamine levels reduce circulating lymphocytes, weakening immune function and increasing SAP risk [31]. Through the cholinergic anti-inflammatory pathway, the PNS releases acetylcholine to inhibit peripheral inflammatory cytokines [32]. Overstimulation of this pathway post-stroke can elevate pulmonary infection risk [33]. In response to post-stroke inflammation, the hypothalamus activates the HPA axis, leading to excessive glucocorticoid secretion [34, 35]. Furthermore, glucocorticoids have anti-inflammatory properties; however, their high levels further suppress immunity, increasing pneumonia risk [36].

This study had several limitations. First, because our study was conducted at a single center and was retrospective, the relatively small sample size might have compromised the power of the primary results. Second, this study used blood parameters on the first day after admission. Therefore, subsequent studies are required to explore the relationship between blood parameters of emergency admission and SAP. Third, whether thrombectomy may cause a systemic inflammatory response to mask the SIRI associated with SAP could not be determined in the present study; therefore, subsequent studies are required to investigate this detail. Finally, Further analysis of the dynamic changes in these inflammatory markers could not be achieved in the present study. Future studies are needed to determine whether changes in the SIRI over time are related to the occurrence of SAP in patients with AIS treated by thrombectomy.

## Conclusions

SIRI is an independent predictor of SAP in patients with AIS treated by thrombectomy. A high SIRI value may contribute to the early identification of patients with AIS treated by thrombectomy at high risk for SAP. Future studies with larger sample sizes are required to confirm these findings.

## Data Availability

The datasets used and/or analysed during the current study are available from the corresponding author on reasonable request.

## Abbreviations

AIS: Ischemic stroke
AUC: Area under the curve
CI: Confidence interval
EVT: Endovascular thrombectomy
GCS: Glasgow Coma Scale
IQR: Interquartile range
NIHSS: National Institute of Health Stroke Scale
OR: Odds Ratio
ROC: Receiver operating characteristic
SAP: Stroke-associated pneumonia
SIRI: Systemic inflammatory response index

## References

[1] Feigin VL, Norrving B, Mensah GA. Global burden of stroke. Circ Res. 2017;120:439–48. 28154096 10.1161/CIRCRESAHA.116.308413

[2] Pinto G, Zétola V, Lange M, Gomes G, Nunes MC, Hirata G, et al. Program to diagnose probability of aspiration pneumonia in patients with ischemic stroke. Int Arch Otorhinolaryngol. 2014;18:244–8. 25992100 10.1055/s-0034-1374646 PMC4297022

[3] Meurer WJ, Barth B, Abraham M, Hoffman JR, Vilke GM, DeMers G. Intravenous recombinant tissue plasminogen activator and ischemic stroke: focused update of 2010 clinical practice advisory from the American Academy of Emergency Medicine. J Emerg Med. 2018;54:723–30. 29545057 10.1016/j.jemermed.2018.01.033

[4] Ganesh A, Goyal M. Thrombectomy for acute ischemic stroke: recent insights and future directions. Curr Neurol Neurosci Rep. 2018;18:59. 30033493 10.1007/s11910-018-0869-8

[5] Smith CJ, Bray BD, Hoffman A, Meisel A, Heuschmann PU, Wolfe CDA, et al. Can a novel clinical risk score improve pneumonia prediction in acute stroke care? A UK multicenter cohort study. J Am Heart Assoc. 2015;4:e001307. 25587017 10.1161/JAHA.114.001307 PMC4330058

[6] Ji R, Shen H, Pan Y, Wang P, Liu G, Wang Y, et al. Novel risk score to predict pneumonia after acute ischemic stroke. Stroke. 2013;44:1303–9. 23482598 10.1161/STROKEAHA.111.000598

[7] Hannawi Y, Hannawi B, Rao CPV, Suarez JI, Bershad EM. Stroke-associated pneumonia: major advances and obstacles. Cerebrovasc Dis. 2013;35:430–43. 23735757 10.1159/000350199

[8] Miller CM, Behrouz R. Impact of infection on stroke morbidity and outcomes. Curr Neurol Neurosci Rep. 2016;16:83. 27485944 10.1007/s11910-016-0679-9

[9] Meisel A, Smith CJ, Stroke. Preventive antibiotics for stroke-associated pneumonia. Nat Rev Neurol. 2015;11:672–3. 26526533 10.1038/nrneurol.2015.220

[10] Li W, He C. Association of platelet-to-lymphocyte ratio with stroke-associated pneumonia in acute ischemic stroke. J Healthc Eng. 2022;2022:1033332. 35340256 10.1155/2022/1033332 PMC8956427

[11] Nam K-W, Kim TJ, Lee JS, Kwon H-M, Lee Y-S, Ko S-B, et al. High neutrophil-to-lymphocyte ratio predicts stroke-associated pneumonia. Stroke. 2018;49:1886–92. 29967014 10.1161/STROKEAHA.118.021228

[12] Cao F, Wan Y, Lei C, Zhong L, Lei H, Sun H, et al. Monocyte-to-lymphocyte ratio as a predictor of stroke-associated pneumonia: a retrospective study-based investigation. Brain Behav. 2021;11:e02141. 33942561 10.1002/brb3.2141 PMC8213641

[13] Kakhki RD, Dehghanei M, ArefNezhad R, Motedayyen H. The predicting role of neutrophil- lymphocyte ratio in patients with acute ischemic and hemorrhagic stroke. J Stroke Cerebrovasc Dis. 2020;29:105233. 33066938 10.1016/j.jstrokecerebrovasdis.2020.105233

[14] Qi Q, Zhuang L, Shen Y, Geng Y, Yu S, Chen H, et al. A novel systemic inflammation response index (SIRI) for predicting the survival of patients with pancreatic cancer after chemotherapy. Cancer. 2016;122:2158–67. 27152949 10.1002/cncr.30057

[15] Yan D, Dai C, Xu R, Huang Q, Ren W. Predictive ability of systemic inflammation response index for the risk of pneumonia in patients with acute ischemic stroke. Gerontology. 2023;69:181–8. 35584610 10.1159/000524759

[16] He Q, Li L, Ren Q. The prognostic value of preoperative systemic inflammatory response index (SIRI) in patients with high-grade glioma and the establishment of a nomogram. Front Oncol. 2021;11:671811. 34055639 10.3389/fonc.2021.671811 PMC8162213

[17] Zhang P, Li Y, Zhang H, Wang X, Dong L, Yan Z et al. Prognostic value of the systemic inflammation response index in patients with aneurismal subarachnoid hemorrhage and a nomogram model construction. Br J Neurosurg. 2020:1–7. 10.1080/02688697.2020.1831438 33044089

[18] Luo F, Li Y, Zhao Y, Sun M, He Q, Wen R, et al. Systemic immune-inflammation index predicts the outcome after aneurysmal subarachnoid hemorrhage. Neurosurg Rev. 2022;45:1607–15. 34718917 10.1007/s10143-021-01681-4

[19] Collins GS, Reitsma JB, Altman DG, Moons KGM. Transparent reporting of a multivariable prediction model for individual prognosis or diagnosis (TRIPOD): the TRIPOD statement. Br J Surg. 2015;102:148–58. 25627261 10.1002/bjs.9736

[20] Ma F, Li L, Xu L, Wu J, Zhang A, Liao J, et al. The relationship between systemic inflammation index, systemic immune-inflammatory index, and inflammatory prognostic index and 90-day outcomes in acute ischemic stroke patients treated with intravenous thrombolysis. J Neuroinflammation. 2023;20:220. 37777768 10.1186/s12974-023-02890-y PMC10543872

[21] Wang R-H, Wen W-X, Jiang Z-P, Du Z-P, Ma Z-H, Lu A-L, et al. The clinical value of neutrophil-to-lymphocyte ratio (NLR), systemic immune-inflammation index (SII), platelet-to-lymphocyte ratio (PLR) and systemic inflammation response index (SIRI) for predicting the occurrence and severity of pneumonia in patients with intracerebral hemorrhage. Front Immunol. 2023;14:1115031. 36860868 10.3389/fimmu.2023.1115031 PMC9969881

[22] Smith CJ, Kishore AK, Vail A, Chamorro A, Garau J, Hopkins SJ, et al. Diagnosis of stroke-associated pneumonia: recommendations from the pneumonia in stroke consensus group. Stroke. 2015;46:2335–40. 26111886 10.1161/STROKEAHA.115.009617

[23] Teh WH, Smith CJ, Barlas RS, Wood AD, Bettencourt-Silva JH, Clark AB, et al. Impact of stroke-associated pneumonia on mortality, length of hospitalization, and functional outcome. Acta Neurol Scand. 2018;138:293–300. 29749062 10.1111/ane.12956

[24] Suda S, Aoki J, Shimoyama T, Suzuki K, Sakamoto Y, Katano T, et al. Stroke-associated infection independently predicts 3-month poor functional outcome and mortality. J Neurol. 2018;265:370–5. 29249057 10.1007/s00415-017-8714-6

[25] Liu D-D, Chu S-F, Chen C, Yang P-F, Chen N-H, He X. Research progress in stroke-induced immunodepression syndrome (SIDS) and stroke-associated pneumonia (SAP). Neurochem Int. 2018;114:42–54. 29317279 10.1016/j.neuint.2018.01.002

[26] C SS. P P, P LS, P RMR. Immunomodulation after ischemic stroke: potential mechanisms and implications for therapy. Volume 20. London, England: Critical care; 2016. 10.1186/s13054-016-1573-1 PMC5141640 27923376

[27] Winklewski PJ, Radkowski M, Demkow U. Cross-talk between the inflammatory response, sympathetic activation and pulmonary infection in the ischemic stroke. J Neuroinflammation. 2014;11:213. 25539803 10.1186/s12974-014-0213-4 PMC4297381

[28] Dorrance AM, Fink G. Effects of stroke on the autonomic nervous system. Compr Physiol. 2015;5:1241–63. 26140717 10.1002/cphy.c140016

[29] Radak D, Resanovic I, Isenovic ER. Changes in hypothalamus-pituitary-adrenal axis following transient ischemic attack. Angiology. 2014;65:723–32. 24065626 10.1177/0003319713503487

[30] Dt NEH. L. The role of neuroendocrine pathways in prognosis after stroke. Expert Rev Neurother. 2014;14. 10.1586/14737175.2014.877841 24428141

[31] K P, C M, C H, J B, E H, T W, et al. Stroke-induced immunodeficiency promotes spontaneous bacterial infections and is mediated by sympathetic activation reversal by poststroke T helper cell type 1-like immunostimulation. J Exp Med. 2003;198. 10.1084/jem.20021098 PMC2194193 12939340

[32] Cheyuo C, Jacob A, Wu R, Zhou M, Coppa GF, Wang P. The parasympathetic nervous system in the quest for stroke therapeutics. J Cereb Blood Flow Metab. 2011;31:1187–95. 21364605 10.1038/jcbfm.2011.24 PMC3099641

[33] Engel O, Akyüz L, da Costa Goncalves AC, Winek K, Dames C, Thielke M, et al. Cholinergic pathway suppresses pulmonary innate immunity facilitating pneumonia after stroke. Stroke. 2015;46:3232–40. 26451017 10.1161/STROKEAHA.115.008989

[34] Offner H, Subramanian S, Parker SM, Afentoulis ME, Vandenbark AA, Hurn PD. Experimental stroke induces massive, rapid activation of the peripheral immune system. J Cereb Blood Flow Metab. 2006;26:654–65. 16121126 10.1038/sj.jcbfm.9600217

[35] Mracsko E, Liesz A, Karcher S, Zorn M, Bari F, Veltkamp R. Differential effects of sympathetic nervous system and hypothalamic-pituitary-adrenal axis on systemic immune cells after severe experimental stroke. Brain Behav Immun. 2014;41:200–9. 24886966 10.1016/j.bbi.2014.05.015

[36] Sundbøll J, Horváth-Puhó E, Schmidt M, Dekkers OM, Christiansen CF, Pedersen L, et al. Preadmission use of glucocorticoids and 30-day mortality after stroke. Stroke. 2016;47:829–35. 26903585 10.1161/STROKEAHA.115.012231

